# Imaging in Patients with Bisphosphonate-Associated Osteonecrosis of the Jaws (MRONJ)

**DOI:** 10.3390/dj4030029

**Published:** 2016-09-02

**Authors:** Britt-Isabelle Berg, Andreas A. Mueller, Marcello Augello, Scott Berg, Claude Jaquiéry

**Affiliations:** 1 Department of Cranio-Maxillofacial Surgery, University Hospital Basel, 4056 Basel, Switzerland; andreas.mueller@usb.ch (A.A.M.); claude.jaquiery@usb.ch (C.J.); 2 Division of Oral and Maxillofacial Radiology, Columbia University Medical Center, New York, NY 10032, USA; 3 Clinic of Cranio-Maxillofacial Surgery, Kantonsspital Aarau, 5001 Aarau, Switzerland; marcello.augello@ksa.ch; 4 Private Practice, 25524 Itzehoe, Germany; scott.berg@radiologie-itzehoe.de

**Keywords:** bisphosphonate-associated osteonecrosis, magnetic resonance imaging, panoramic radiograph, computed tomography, cone beam computed tomography, single photon emission computed tomography, positron emission tomography, fluorescence-guided bone resection

## Abstract

Background: Bisphosphonate-associated osteonecrosis of the jaws (MRONJ/BP-ONJ/BRONJ) is a commonly seen disease. During recent decades, major advances in diagnostics have occurred. Once the clinical picture shows typical MRONJ features, imaging is necessary to determine the size of the lesion. Exposed bone is not always painful, therefore a thorough clinical examination and radiological imaging are essential when MRONJ is suspected. Methods: In this paper we will present the latest clinical update on the imaging options in regard to MRONJ: X-ray/Panoramic Radiograph, Cone Beam Computed Tomography (CBCT) and Computed Tomography (CT), Magnetic Resonance Imaging (MRI), Nuclear Imaging, Fluorescence-Guided Bone Resection. Conclusion: Which image modality is chosen depends not only on the surgeon’s/practitioner’s preference but also on the available imaging modalities. A three-dimensional imaging modality is desirable, and in severe cases necessary, for extended resections and planning of reconstruction.

## 1. Introduction

Bisphosphonate-associated osteonecrosis of the jaws is a commonly seen disease in cranio-maxillofacial surgery, first described by Marx in 2003 [1]. Marx did not use an abbreviation for this disease in his “letter to the editor” [1] but in an increasing number of articles the following can be found: MRONJ, BP-ONJ, and BRONJ. Based on the updated position paper of the American Association of Oral and Maxillofacial Surgeons (AAMOS), in this article their staging system and the abbreviation “MRONJ” will be used [2]. In quotations, the abbreviation of the author(s) is used. The prevalence of MRONJ in patients with intravenous treatment and a malignant primary disease is higher than in patients with benign diseases. Prevalence rates vary [3,4,5] and can be as high as 21% in malignant diseases [4]. The aetiology of MRONJ is still not fully understood in detail but several factors might increase the risk. This includes high dosages of bisphosphonates, invasive surgical procedures, possibly periodontal diseases, and the half-life of bisphosphonates [6,7]. “The half-life of bisphosphonates in bone is very long, ranging among different species from 1 to 10 years, depending largely on the rate of bone turnover” [8]. Since constant remodelling of the jaws bone occurs, application of bisphosphonate treatment will lead to osteoclast inhibition on the one hand, and on the other hand to new bone formation by osteoblasts [9]. During this process, bisphosphonates will accumulate in the bone. Other have aspects have been reported, such as “soft-tissue toxicity”, of bisphosphonates leading to the “presence of micro-abscesses and intense inflammatory infiltrate in the hypoderm permeating the muscle fibres and fat lobules” [10]. Once a patient suffers from a MRONJ, typical findings are necrotic (alveolar) bone, inflammation of the surrounding tissues (in some cases), and fistulas. Depending on inflammation and the involvement of nerve structures, MRONJ is often painful and will reduce the quality of life. Therefore, it is important to try to prevent MRONJ. Once the clinical picture shows typical MRONJ features, imaging is necessary to determine the size of the lesion. Exposed bone is not always painful [11,12], therefore a thorough clinical examination and radiological imaging are essential when MRONJ is suspected. In this paper we will present the latest clinical update on the imaging options in regard to MRONJ.

## 2. Anatomical Imaging

Morphological anatomical imaging including panoramic radiographs, cone-beam computed tomography (CBCT), computed tomography (CT), and magnetic resonance imaging (MRI) will be discussed in this section. Functional nuclear imaging techniques and fluorescence will be discussed in the subsequent section.

### 2.1. Panoramic Radiographs

**Figure 1:**
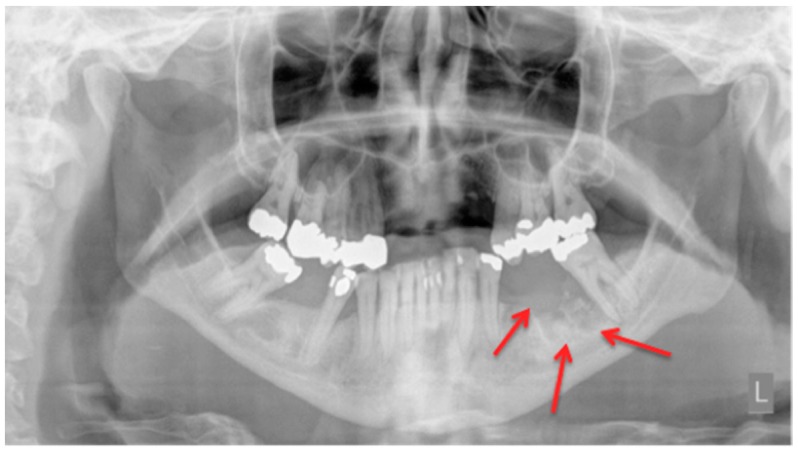
Panoramic radiograph: Patient: 48 years old, female, metastatic breast cancer, zoledronic acid. **Red** arrows point to the necrotic area. The corresponding magnetic resonance imaging (MRI) images are shown in Figure 6 and Figure 7. American Association of Oral and Maxillofacial Surgeons (AAMOS) staging: stage 2.

**Figure 2:**
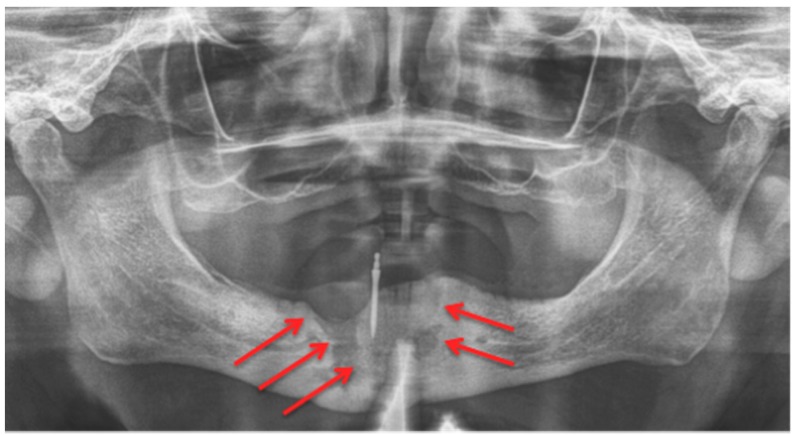
Panoramic radiograph: Patient: 77 years old, male, metastatic prostate cancer, ibandronic acid & later another antiresorptive drug: denosumab. **Red** arrows point to the necrotic area. Artefact due to thyroid shield. For the corresponding cone-beam computed tomography (CBCT) image, see Figure 4. AAMOS staging: stage 2.

**Figure 3:**
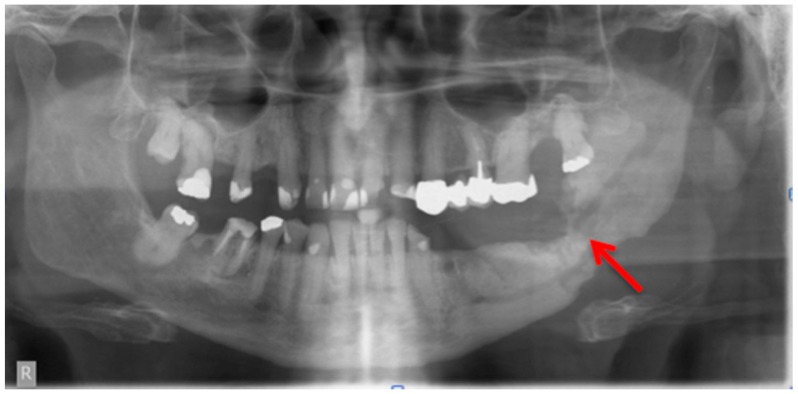
Panoramic radiograph: Patient: 66 years old, male, secondary osteoporosis due to castration, alendronate. **Red** arrow indicates the almost invisible fracture. The computed tomography (CT) scan is shown in Figure 6 and the corresponding single photon emission computed tomography (SPECT) and SPECT/CT images in Figure 8 and Figure 9 respectively. AAMOS staging: stage 3.

In a daily routine, a clinical examination and a radiograph are the minimum examinations required in order to detect lesions and to provide data for a follow-up appointment. For Marx et al., panoramic imaging is the image of choice for a routine dental assessment in these patients [13]. Figure 1, Figure 2 and Figure 3 exemplarily depict typical findings of panoramic radiographs.

Rocha et al. were able to show that patients who “are treated with zoledronate presented a statistically significant increase in the number of radiographic abnormalities compared with the control group” [14]. Although their sample size was not very large and only 30 patients were treated with zoledronic acid early bone alteration was found in these patients. As stated by Arce et al., conventional anatomic imaging is easily accessible but bone changes and radiographic findings can have a lag time of up to two weeks [15]. Phal et al. found in their study that all patients showed osseous sclerosis. The alveolar margin was involved in two-thirds of the patients. Lamina dura thickening, full-thickness sclerosis, poor/non-healing extraction sockets, widening of the periodontal ligament space, osteolysis, as well as sequestra, fistula, soft tissue thickening, and periosteal new bone formation were also found and described [12]. Patients who received follow-up imaging showed progressive sclerotic changes leading to possible narrowing of the mandible canal [12]. A study published by Torres et al. was able to show that in panoramic radiographs the “mean mandibular inferior cortical bone thickness (MICBT) of patients with BRONJ was significantly higher compared to patients without BRONJ taking BPs, and mean MICBT of patients without BRONJ taking BPs was higher than that of controls”. They could also show that “among patients taking zolendronate, there was a correlation between MICBT and cumulative dose” [16]. An animal model (rats) also showed changes in the bone after animals were treated with a zoledronic acid and dexamethasone followed by extraction of mandibular or maxillary molars. The radiographs from these animals showed “poor definition of the alveolar ridge with mixed radiodensity” [17]. A prospective study from Stockmann et al. revealed detectability of MRONJ lesions was 54% for panoramic radiographs, 96% for CT, and 92% for MRI scans [18]. Their study acquired all images 10 days prior to surgery. The images were assessed by two experienced radiologists who were unaware of the clinical findings. Panoramic radiographs were only evaluated in regard to possible identification of all jaw regions affected by MRONJ [18]. Treister et al. stated that they found a “trend towards higher stage with greater radiographic findings”, but it was not significant [19].

Another aspect of radiograph use was assessed and discussed by Walter et al. [6]. They retrospectively analysed 129 panoramic radiographs, which were acquired at patients’ first appointments. For each MRONJ patient, a control patient was selected. This control had matching age and gender. In X-rays they found nearly twice as much bone loss among the remaining teeth for the MRONJ group in comparison to the control group. Unfortunately, no clinical probing values were recorded. Nevertheless, this study assessed periodontal disease and possibly linked it to MRONJ [6]. Bianchi et al. published a study comparing panoramic radiographs with CT scans. Their results showed that 3D imaging (CT) was superior [20]. The follow-up by Bedogni et al.—who, in their study, obtained panoramic radiographs and CT scans at 3, 6, 12, 18, and 24 months [21]—does not conform with the “ALAR” guidelines of the “European guidelines on radiation protection in dental radiology” [22].

To sum up, we agree with Stockmann et al.’s conclusion: “even if BONJ lesions can be detected on panoramic radiographs, an adequate assessment of the extent of BONJ is not possible” [18], and therefore panoramic imaging is usable, but in severe cases must be followed by further diagnostics.

### 2.2. Cone-Beam Computed Tomography

**Figure 4:**
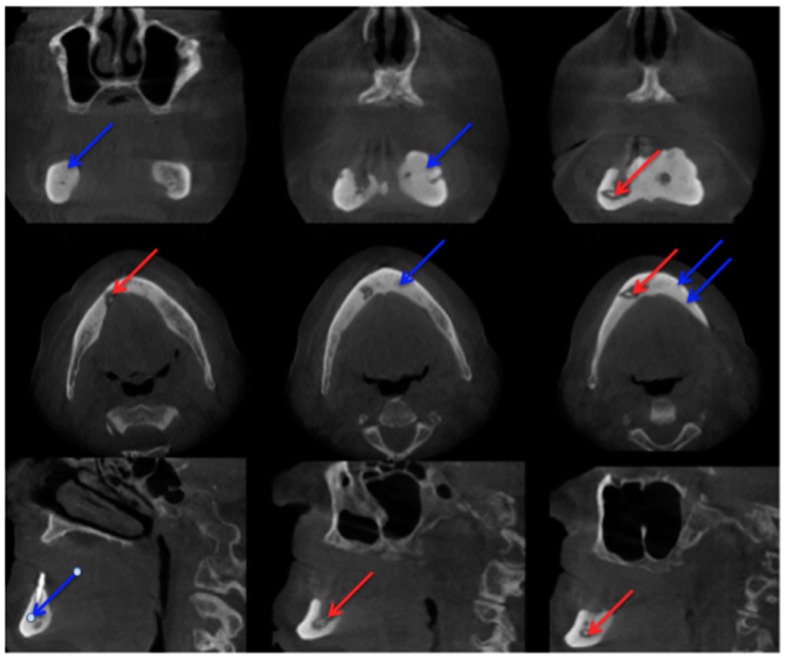
Cone-beam computed tomography (Carestream CS 9300) Patient: 77 years old, metastatic prostate cancer, ibandronic acid and later another antiresorptive drug: denosumab. For panoramic radiograph see Figure 2. 1st row: coronary view; 2nd row: axial view; and 3rd row: sagittal view. **Red** arrows: sequester; **blue** arrows: sclerotic region. AAMOS staging: stage 2.

In comparison with MRI and CT, there are fewer papers about imaging of MRONJ with CBCT [23]. Before Torres et al. investigated mandibular inferior cortical bone thickness [16], they studied cortical bone dimensional changes in MRONJ patients using CBCT data sets. By assessing the data sets (12 patients/66 test persons) using three different techniques, they could show that “the cortical bone measurements were significantly higher in cases than controls for all 3 techniques” [24]. As stated in Yalcin and Gungormu’s review, typical findings in CBCT and CT are “pathologic fractures, narrowing of the marrow space and involvement of the inferior alveolar canal” [23]. Figure 4 shows a patient with narrowing of the marrow space and sequestra from our clinical database.

Detecting periosteal thickening or bone density changes at an early stage before it gains clinical importance might be another case [23,25] for the use of CBCT. Wilde et al. stated that the two most common findings in CBCT for MRONJ are “destruction of the trabecular structure of the cancellous bone and erosion of the cortical bone” [26]. The authors retrospectively assessed 27 CBCT scans from MRONJ patients, and aspects of “sclerotic manifestations” were discussed. Contrary to Olutayo et al. [27] they did not find a correlation between sclerotic manifestation and the severity of the MRONJ [26]. Treister et al. described CBCT as superior at detecting fragmentation and sequestra in comparison to panoramic radiographs [28]. In our examples (Figure 1 panoramic radiograph and Figure 4 CBCT scan) the extent is visible in both modalities but more detectable in the CBCT scan. Guggenberger et al. found that CBCT imaging and clinical examination showed less extensive changes than positron emission tomography (PET)/CT and MRI [29]. A model using CBCT scans was developed by Barragan-Adjemian et al. [30]. Based on their observations, they found an explanation of how an exposed sequestra develops. In detail, their model describes, “the formation of a necrotic body (or bodies) or involucrum(s) inside the trabeculae in sclerotic mandibular bone. The involucrum most likely represents dead bone that becomes surrounded by a resorptive circumference that increases with time.” [30]. Additionally, “the involucrum follows the path of least resistance (which is the easiest and quickest means to remove the necrotic body either lingual or towards the edentulous area) leading to an exposed sequestrum (clinical ONJ) or, alternatively, if the tooth is missing moves towards the edentulous area.” [30]. Since Cankaya et al. [31] found in their rat model that “the extent of the BONJ lesions assessed from CBCT scans did not differ significantly from the intraoperative situation, and a significant correlation between CBCT measurements and intraoperative measurements was found” [31], and radiation dosage a low as just 3 μSv (effective dose 5 × 5 cm adult exam) are commercially published [32], CBCT might gain even greater relevance in the future.

### 2.3. Computed Tomography

**Figure 5:**
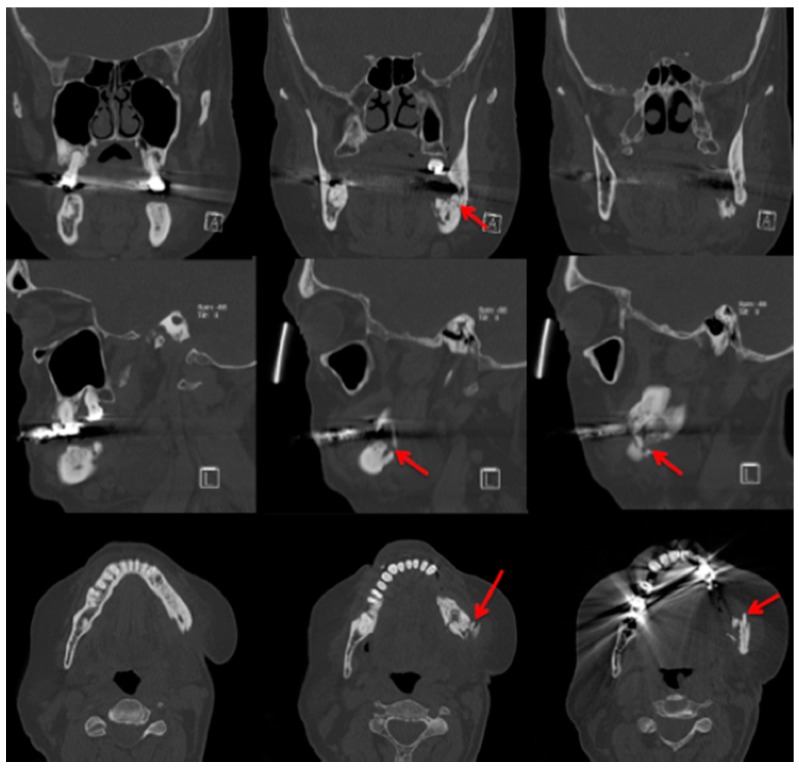
Computed tomography (Siemens, Sensation 64) Patient: 66 years old, male, secondary osteoporosis due to castration, alendronate. Red arrow points in the direction of the fracture due to the bisphosphonate necrosis. The panoramic radiograph is shown in Figure 3 and the 99Tcm-DPD SPECT and 99Tcm-DPD SPECT/CT in Figure 8 and Figure 9 respectively. AAMOS staging: stage 3.

Bianchi et al. [20] assessed 32 panoramic radiographs and CT scans in detail for the following features: “structural alteration of trabecular bone, from initial change in thickness and mineral content of the trabeculae to the formation of microlacunae; cortical bone erosion; osteosclerosis; small (less than 15 mm) sequestrum; extensive (more than 15 mm) sequestrum; and presence of periosteal new bone.” They found that CT was superior to dental panoramic radiographs in detecting all the radiologic signs [20]. Cortical bone erosion and trabecular bone resorption were visible to different extents. In their readings they found that panoramic radiographs missed the correct diagnosis of sequestra in 15 cases [20]. In our example case, even a fracture could be missed if only the panoramic radiograph is assessed (Figure 3 and Figure 5).

The extended follow-up (CT scans at 3, 6, 12, 18, and 24 months) by Bedogni et al. was able to show that “CT signs of recurrent disease are apparent within 6 months after surgery and precede clinical manifestations of BRONJ” [21]. Sanna et al. stated that CT helps to differentiate between MRONJ and metastasis [33]. Elad et al. assessed 110 CT scans and stated “the mandibular canal cortex was resistant to the destructive process of the jaw, unlike in metastases” [34], but there are MRONJ cases which are still difficult to diagnose even with CT. Clinical examination is mandatory for diagnosis [35]. Farias et al. mentioned that the CT image features did not differ significantly between cases with or without exposed bone [35]. Not only are changes in bone investigated in patients with MRONJ, but thickening of the sinus maxillary mucosa is also examined [20]. A study by Gallego et al. were able to show patients with MRONJ had greater probability of presenting sinus mucosal thickening in comparison to a healthy group. They used a thickening of >3 mm as their measurement value. In their assessment, they found that the thickening was present more in patients with “advanced-stage disease” [36]. In their study, Hutchinson et al. concentrated on radiographic characteristics in patients with “stage 0 disease” [37]. Ten patients met the criteria for “stage 0”. They included 1× panoramic image, 2× CBCT scans, and 9 CT scans. The authors reported “Diffuse osteosclerosis in clinically symptomatic areas, characterised most with extension beyond the involved site, density confluence of cortical and cancellous bone, prominence of the inferior alveolar nerve canal, markedly thickened and sclerotic lamina dura, uniform periradicular radiolucencies, cortical disruption, lack of bone fill after extraction, and a persisting alveolar socket.” [37]. Hamada et al. [38] reported “A simple evaluation method for early detection of bisphosphonate-related osteonecrosis of the mandible using computed tomography”. They found no significant differences between means of administration (intravenous or oral) and length in regard to “cancellous bone CT radiodensity value, cortical bone CT radiodensity value, and cortical bone width.” [38]. Otherwise they found differences between the MRONJ group and a control group regarding cortical bone width. In cancellous bone, CT radiodensity values even varied “among the BRONJ area, the non-BRONJ area, and the control groups” [38]. A limitation of this study, which should be mentioned, was the size of the MRONJ group. A total of 20 patients were included; only 4 patients received bisphosphonates orally. Of these 4 patients, 3 patients received risedronate and 1 patient received alendronate [38], therefore although it provides some valuable information, a larger sample size would be desirable. The patient group assessed in the article by Wutzl et al. included 17 patients. All of them presented sclerotic zones. In 9 of these patients MRI and scintigraphy were performed. They stated that, “sclerotic changes on the CT scan appeared hypointense on MRI.” [39]. Bisdas et al. found “sclerotic regions in the jaws with or without periosteal bone proliferation.” [40]. A large multicentre study (“MISSION”) assessed in detail 799 patients suffering from MRONJ in regard to staging/CT. From their point of view, future staging systems should consider both clinical signs and CT findings [41].

### 2.4. Magnetic Resonance Imaging

**Figure 6:**
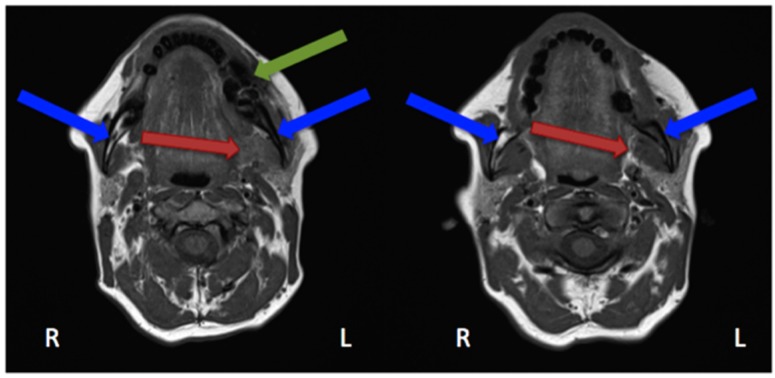
Magnetic resonance imaging (Siemens, Avanto, 1.5T, Sequence: T1 tse tra) Patient: 48 years old, female, metastatic breast cancer, zoledronic acid for 2 years (panoramic radiograph Figure 1). **Green** arrow showing the MRONJ necrosis, **red** arrows showing the oedema, **blue** arrows showing the differences between the right side: normal fatty bone marrow and left side: signal loss, due to loss of fat. Pair of screenshots. AAMOS staging: stage 2.

**Figure 7:**
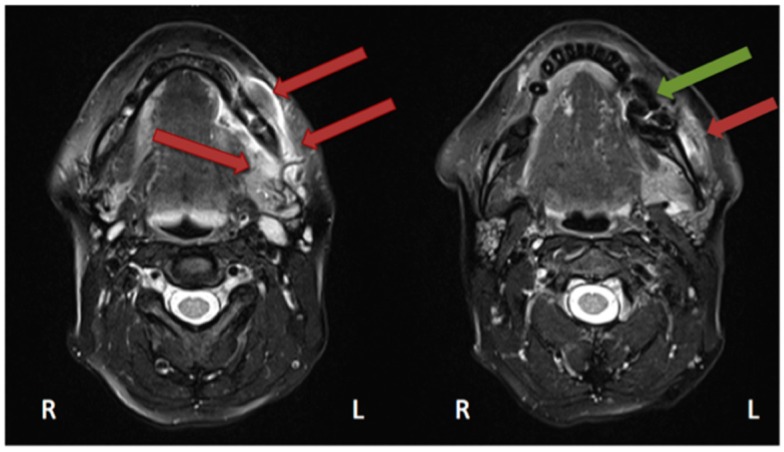
Magnetic resonance imaging (Siemens, Avanto, 1.5T, Sequence: T2 tse tra) Patient: 48 years old, female, metastatic breast cancer, zoledronic acid for 2 years (panoramic radiograph Figure 1). **Green** arrow showing the MRONJ necrosis: hypointense bone marrow, **red** arrows showing the oedema. Pair of screenshots. AAMOS staging: stage 2.

In the study by Guggenberger et al., all MRONJ foci “showed markedly decreased signal on T1-with increased signal on T2-weighted images” [29]. This was the case for all except one patient, in whom an intermediate signal on T2 was seen. “Contrast uptake of affected bone and surrounding tissue was noted in all patients and foci of BONJ” [29]. Furthermore, the authors described that contrast-enhanced MR imaging shows more extensive changes in comparison with the clinical examination and CBCT imaging [29]. In their study, Stockmann et al. stated that “MRI has a high detectability for BONJ lesions” [18] but limitations were found concerning the extent of the detection [18]. Bedogni et al. assessed in their study 11 MRI scans performed on MRONJ patients [42]. Gadolinium (intravenous) was used as a contrast agent. These images showed two patterns of bone disease: “Exposed areas showed a low signal in T1- and T2-weighted and inversion recovery images, which suggests low water content and is histopathologically correlated with paucity in cells and vessels (osteonecrotic pattern). Unexposed diseased bone was characterised by T1 hypointensity and T2 and IR hyperintensity, which suggests high water content and inflammation, associated with hypercellularity, osteogenesis, and hypervascularity (osteomyelitic pattern).” [42]. Hypointensity in T1 was also seen in MRI scans performed on our patient (Figure 6 and Figure 7).

Krishnan et al. described early MRI findings of MRONJ in their publication. This includes at the early stage “the loss of the normal T1 hyperintensity of fatty marrow in the mandible and maxilla.” [43] “Bone destruction, soft tissue edema and enhancement, inferior alveolar nerve thickening, and pterygoid muscle swelling and enhancement” [43] are findings of more advanced stages. Exemplary MRI protocols are found in the publications by Bedogni et al., Stockmann et al. or García-Ferrer et al. [18,42,44]. Although MRI scans are very helpful for defining the extent, the 99Tcm-MDP 3-phase bone scan was superior to both CT and MRI [45].

## 3. Functional Imaging

Functional imaging, as described in the paragraph above, is gaining increasing importance. Functional imaging on its own in combination with anatomical imaging is described below.

### 3.1. 99Tcm-DPD/99Tcm-MDP Bone Scan/Scintigraphy/Single Photon Emission Computed Tomography/Computed Tomography (SPECT/CT)

In functional imaging of bone diseases, a skeletal scintigraphy is one of the basic imaging modalities (“bone scan”). In a case analysis by Chiu et al. 10 out of 13 patients showed focal abnormal activity (increased radionuclide uptake with central decrease) in scintigraphy imaging [46].

**Figure 8:**
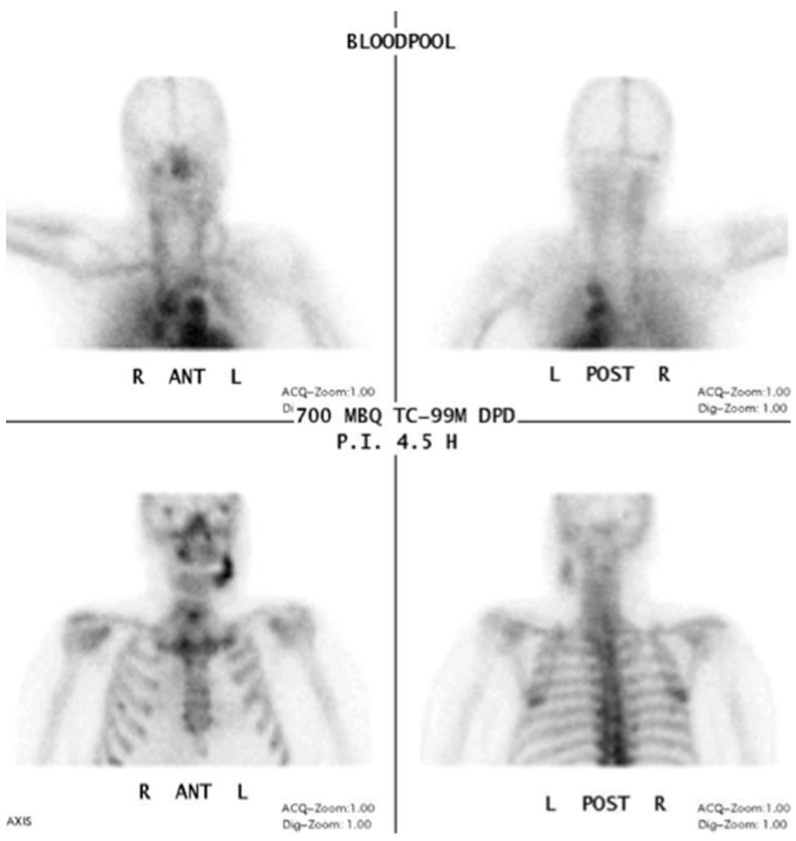
Planar scintigraphy (Siemens, Symbia) Blood pool phase. Patient: 66 years old, male, secondary osteoporosis due to castration, alendronate. AAMOS staging: stage 3.

**Figure 9:**
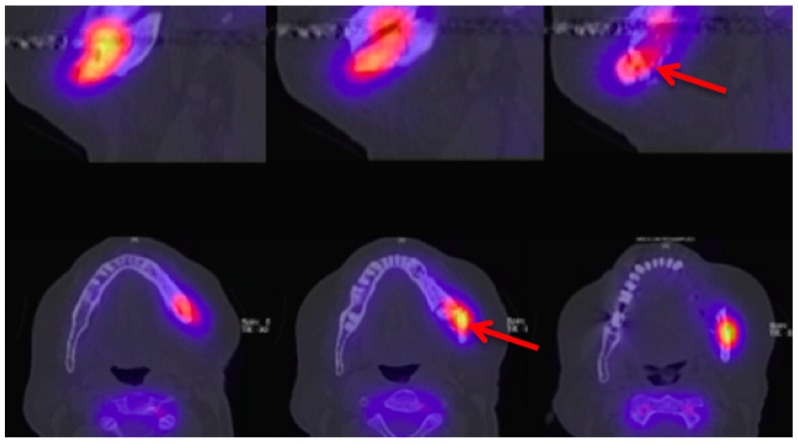
Technetium-99m-3,3-diphosphono-1,2-propanodicarboxylicacid (99Tcm-DPD) SPECT/CT (Siemens, Symbia) Patient: 66 years old, male, secondary osteoporosis due to castration, alendronate. First row sagittal, second row axial view; 4.5 h after injection (bone phase). The uptake in the **left** mandible is clearly visible (**red** arrow). For the panoramic radiograph see Figure 3. AAMOS staging: stage 3.

Information from a planar scintigraphy is displayed in a two-dimensional form in contrast to SPECT. An example of technetium-99m-3,3-diphosphono-1,2-propanodicarboxylicacid (99Tcm-DPD) SPECT and 99Tcm-DPD SPECT/CT is seen in Figure 8 and Figure 9 respectively.

In SPECT imaging, the distribution of the radionuclide is monitored in multiple two-dimensional images and from multiple angles. From these datasets, a three-dimensional image is then calculated. If anatomical imaging should be added, hybrid SPECT/CT scanners are available. For detecting bony infections, technetium-99m methylene diphosphonate (99Tcm-MDP) or technetium-99 m-DPD (99Tcm-DPD) are frequently used nuclides; they show no significant differences in detecting “pathologically increased bone uptake” [47]. MRONJ should not show an uptake in the necrotic zone, but due to the associated infection, a nuclide uptake may be seen. O’Ryan et al. [48] published a retrospective study on MRONJ patients who had received whole-body planar bone scintigraphy. They used the following scoring system for the jaw: “score 0, no visual evidence of increased uptake was present; score 1, uptake was mild and equal to that in the sternum; and score 2, uptake was intense and greater than that in the sternum” [48]. The comparison with the sternum uptake was based on a paper published by Kakhki et al. [49]. Kakhki et al.’s paper, a study on 334 patients who had no diseases of the sternum/chest wall or malignancy, assessed the normal uptake in a sternum considering the age of the patient [49]. O’Ryan et al. found a nuclide uptake in 65.7% of their MRONJ patients. From their point of view, scintigraphy might have a prognostic value and physicians should watch out for newly occurring uptake in the jaws [48]. Thomas et al. assessed the impact of bone scintigraphy in patients with metastatic castration-resistant prostate cancer who had received bisphosphonates. Their focus was on early prediction of clinically asymptomatic MRONJ. MRONJ was significantly more often developed in patients with a pathological tracer uptake [50]. Ristow et al. investigated the bone turnover in the jaw of breast cancer patients who had received no antiresorptive medication, bisphosphonates, or denosumab. Interestingly, they found that “there was similar turnover of bone in the mandible compared with other skeletal sites (such as the femur), while the maxilla showed significantly higher turnover”. Since the majority of MRONJ lesions occur in the mandible, the bone turnover role of the MRONJ pathogenesis must be further reviewed [51].

### 3.2. 18F-FDG Positron Emission Tomography/Computed Tomography (PET/CT)

Fludeoxyglucose F18 (18F-FDG) PET/CT combines anatomical imaging and functional imaging as described previously in SPECT/CT. In comparison to PET imaging, it can be expected that infected bone tissue will show increased glucose metabolism, an increased uptake in comparison to necrotic areas. Therefore, why use imaging in suspected necrotic areas where no blood flow and hypermetabolism occurs [52]? “Abnormal mandibular enhancement on PET scan is not necessarily an indicator of MRONJ, but rather a reflection of an inflammatory process.” [52]. If this imaging is available, early detection and assessment of an inflammatory process, which is one of the risk factors for the development of a MRONJ, might be possible. This might prevent progression of the disease. Fleisher et al. retrospectively analysed the PET/CT scans from 23 patients (treated with bisphosphonate and/or denosumab) and concluded that PET/CT can be a helpful tool since their results showed that “FDG PET/CT detects local and diffuse metabolic changes that may not be represented by plain radiography” [53]. For evaluation a normal reference has to be taken: “normal bone of the contralateral side of the jaw, the cervical vertebrae, and the skull base as the normal reference.” [29]. Conclusively we support Fleisher et al.’s statement that “PET imaging cannot identify osteonecrosis that is not associated with infection (i.e., aseptic necrosis) or a reactive or reparative process” [53].

### 3.3. Fluorescence-Guided Bone Resection/Visually Enhanced Lesion Scope (VELscope^®^)

Fluorescence-guided bone resection is a precisely described way of imaging in combination with surgery in MRONJ patients. The following method is published: pre-operatively, the patient receives 100 mg doxycycline twice a day for 10 days. The viable bone will have a doxycycline uptake and will present a “greenish” light when illuminated by the VELscope^®^ (LED Dental, White Rock, BC, Canada). The fluorescence of vivid bone will be visualised “under blue excitation light of 400 to 460 nm” [54] and “it should be noted that the green fluorescent filter fitted to the handpiece is an essential component that separates the doxycycline fluorescence from the bright exciting light of the lamp” [54]. Necrotic bone will not have an uptake therefore no/very little fluorescence is shown. In a study by Pautke et al., “bleeding of the bone during resection did not correlate with any bone fluorescence signal” [55]. In cancellous bone regions, bone bleeding can occur, suggesting viable bone, but fluorescence is not seen [54]. This technique might offer a way to standardise the surgical procedure [55]. In a study performed by Assaf et al., 20 patients were included in a prospective study. Except in one case, necrotic lesions were visible using the VELscope^®^. Even in a patient who received only a single 100 mg shot of doxycycline one hour pre-operatively, it was possible to distinguish between necrotic and healthy bone using the VELscope^®^ [56]. Based on these findings and their own observations, Ristow and Pautke published a study on 8 patients using auto-fluorescence of healthy bone without doxycycline/tetracycline labelling. Using the VELscope^®^ Vx, vivid bone showed an auto-fluorescence. The bone history of all samples showed removal of the necrotic bone [57].

## 4. Conclusions

Which image modality is chosen depends not only on the surgeon’s/practitioner’s preference but also on the available imaging modalities. A three-dimensional imaging modality is desirable, and in severe cases necessary, for extended resections and planning of reconstruction. CT imaging is commonly available and assessment of the soft tissue is possible in comparison to CBCT. CBCT is also a valuable imaging modality due to the lower radiation in comparison with a spiral CT scan. Functional imaging is useful for detection of MRONJ at an early stage, especially if routine nuclear imaging is acquired for metastasis search or follow-up purposes, but also to capture lesions which are not obvious on anatomical imaging. The use of the VELscope^®^ proved to be very helpful to detect the extent of the necrosis more precisely, and its use in the operation theatre is highly feasible and not time-consuming.
